# Unidirectional barbed sutures vs. interrupted intracorporeal knots in thoracoscopic repair of congenital diaphragmatic hernia in pediatrics

**DOI:** 10.3389/fped.2024.1348753

**Published:** 2024-01-18

**Authors:** Mohamed Ali Shehata, Mohamed Ahmed Negm, Mohamed Mahmoud Shalaby, Mohamed Awad Mansour, Ahmed Abdelmhaimen Elhaddad

**Affiliations:** ^1^ Pediatric Surgery Department, Faculty of Medicine, Tanta University, Tanta, Egypt; ^2^ Pediatric Surgery Unit, Qena Faculty of Medicine, South Valley University, Qena, Egypt

**Keywords:** unidirectional barbed sutures, interrupted intracorporeal knots, thoracoscopy, congenital diaphragmatic hernia, pediatrics

## Abstract

**Background:**

Intracorporeal suturing knots continue to be one of the most challenging and time-consuming steps in the thoracoscopic repair of congenital diaphragmatic hernia (CDH). Barbed unidirectional knotless sutures are designed to shorten surgical procedures by eliminating the need to tie knots. This work aimed to compare unidirectional barbed sutures and interrupted intracorporeal knots in the thoracoscopic repair of CDH in pediatrics regarding the time required to suture, operative time and complications.

**Methods:**

This retrospective study included 139 patients presented with Bochdalek CDH. Patients were classified into early (neonatal) and late presentations. The hernia defect was repaired by unidirectional **B**arbed sutures (V-Loc^TM^ and Stratafix^TM^ sutures) in group **B** or by **C**onventional interrupted intracorporeal knots in group **C**.

**Results:**

In both early and delayed presentations, the time required to suture (15 and 13 min in group B, 33 and 28 min in group C for neonatal and delayed presentation respectively) was significantly shorter in group B. Complications (visceral perforation, wound infection, and recurrence) insignificantly differed between group B and group C of early presentation. No patients suffered from major complications in both groups.

**Conclusions:**

Both unidirectional barbed sutures and intracorporeal knots were safe and effective. However, unidirectional barbed sutures are a time-saving choices for CDH thoracoscopic repair in early and late presentations.

## Introduction

Congenital diaphragmatic hernia (CDH) is a problem in the development of the diaphragm that affects roughly 1 in 2,500 neonates (1).

In newborns and children, minimally invasive surgery (MIS) was adopted years ago, following its introduction in adults. This method has evolved from less invasive diagnostic procedures to more sophisticated treatment interventions (2).

MIS CDH repairs have been done, either laparoscopic or thoracoscopic. In 1995, the first pediatric thoracoscopic repair was recorded. The Congenital Diaphragmatic Hernia Study Group (CDHSG) in 1997 revealed that subcostal laparotomy is the most prevalent method for repair (91%) (3). In 2001, a report on thoracoscopic correction of CDH infants with delayed presentation was published. Then, neonatal thoracoscopic repair was introduced (4).

Intracorporeal suturing continues to be one of the most challenging and time-consuming steps resulting in extending the duration of the operation. Prolonged contact with CO_2_ and anesthesia may increase morbidity risk, especially in neonates (5).

Barbed unidirectional knotless sutures (V-Loc^TM^ and Stratafix^TM^ sutures) are designed to reduce the operation time by eliminating the necessity to tie knots. Barbed suture anchoring inhibits migration and can be considered a “continuously interrupted” suture void of knots. It has been established that these sutures have at least the same tissue-holding functioning as traditional sutures (6). The knotless, self-anchoring design permits simple tissue approximation with less time and cost (7). These FDA-approved sutures are used for soft tissue repair and are broadly applied through MIS urology, gynecology, orthopedic and plastic procedures (7–11).

In children, barbed sutures were demonstrated to be effective, safe, and timesaving in gastrointestinal MIS (12). However, there is no report evaluating the efficacy and safety of barbed sutures in early and late presentation children undergoing thoracoscopic surgery for CDH. Therefore, this work aimed to compare unidirectional barbed sutures and interrupted intracorporeal knots in the thoracoscopic repair of CDH in pediatrics regarding the time required to suture, operative time and complications.

## Methods

This retrospective study involved 139 pediatrics of both sexes presented with Bochdalek CDH. The research was conducted with the approval of the Research Ethics Committee, Faculty of Medicine, Tanta University, Egypt (approval code: 36264PR27/1/23). Informed written consent was obtained from all patient's guardians. The patients' data were collected from January 2018 to December 2022.

Exclusion criteria were central and para-esophageal CDH, need for a patch, arterial blood gas problem (PaCO_2_ > 60 mmHg, pH < 7.25) which was not alleviated by conventional ventilation, unstable cardiovascular and respiratory status who require mechanical ventilation, preterm infants, persistent pulmonary hypertension of the newborn, associated severe malformations (particularly complex cardiac abnormalities), eventration of the diaphragm, and acute intestinal obstruction with or without suspected strangulation.

Patients were classified into early (neonatal) presentation and late presentation. Patients were subdivided into two groups based on the type of sutures used to repair the hernia by closed envelop method. The hernia defect was repaired by unidirectional Barbed sutures (V-Loc^TM^ and Stratafix^TM^ sutures) in group B or by Conventional interrupted intracorporeal knots in group C. Operations were done by two surgeons both are expert in thoracoscopic repair of congenital diaphragmatic hernia and using barbed sutures.

**Figure 1:**
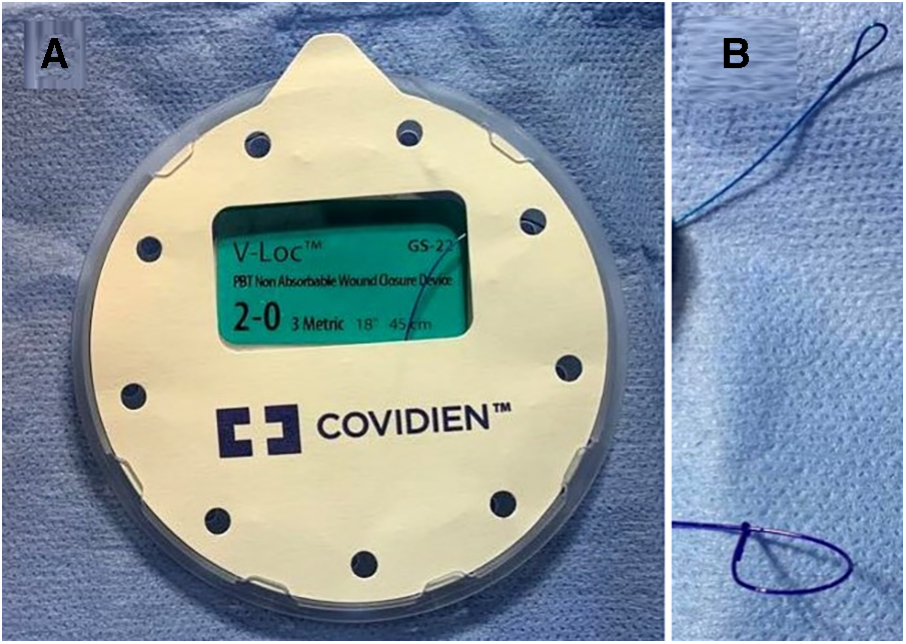
(**A**) V-loc^TM^ sutures pack; (**B**) upper is end (loop) of V-loc^TM^ suture and lower of stratafix^TM^.

**Figure 2:**
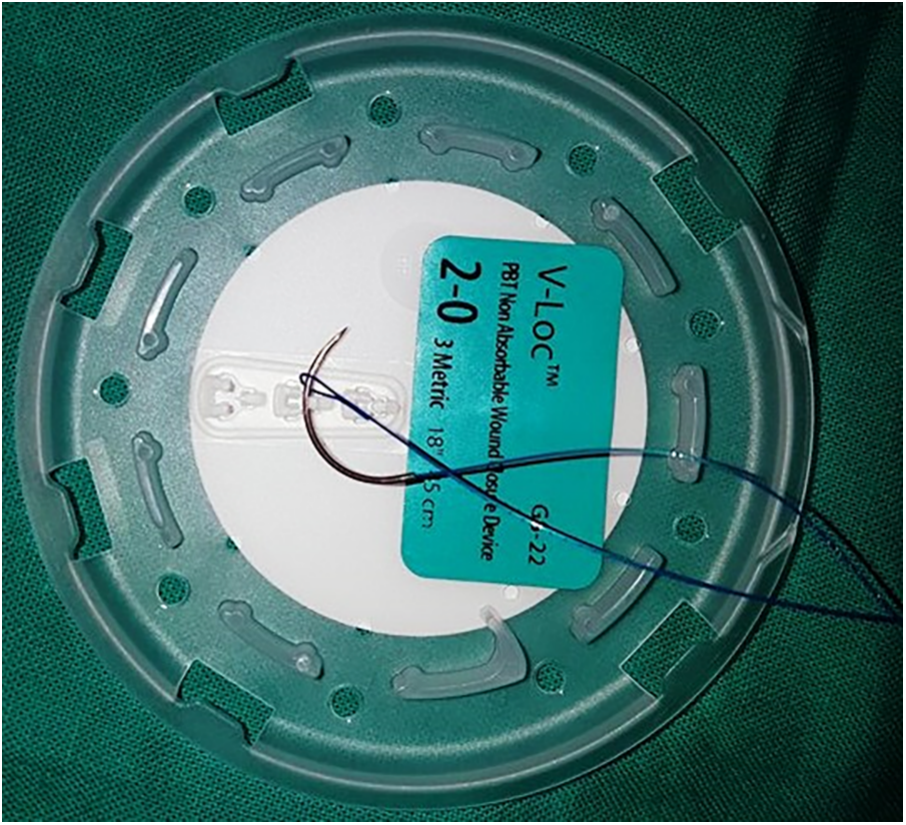
V-Loc^TM^ sutures needle passed through the loop at the end of suture (to make the first knot).

Sutures used in group B are permanent 3/0 or 2/0 according to baby age, on a round needle, taper point, half curvature, length 17 or 26 mm (types of needles are RB-1 and CT-2 in Stratafix^TM^, and CV-23 and GS-22 in V-Loc^TM^) (Figures 1, 2). In group **C** interrupted non-absorbable suture, mostly Ethibond^TM^ braided non-absorbable suture 3/0 or 2/0 according to baby age, on a round needle, taper point, half curvature, length 17 or 26 mm.

Preoperative data of the studied patients were retrieved, including age, sex, weight, side, presence of sac and hernia contents.

### Technique

**Figure 3:**
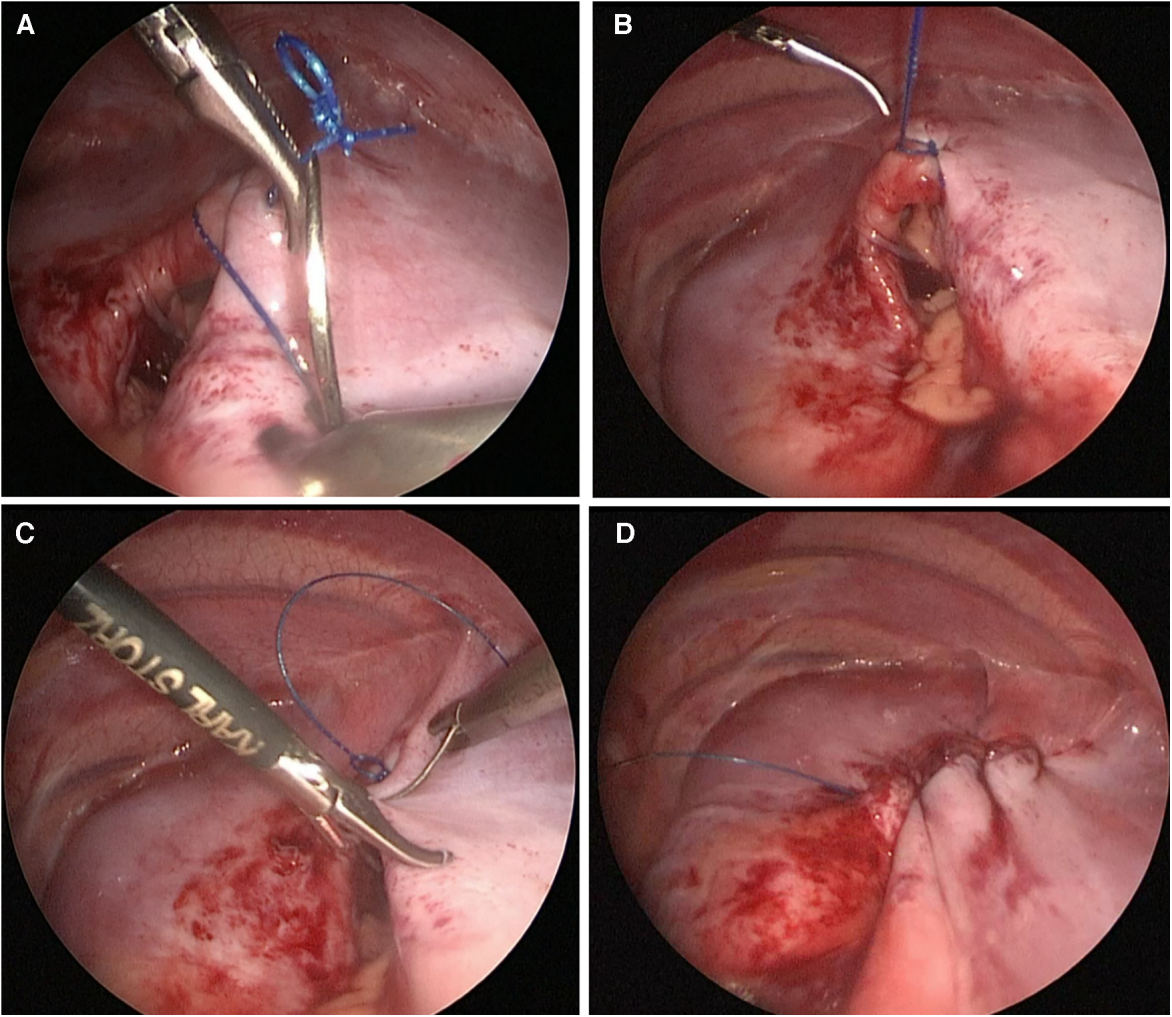
Repair of the diaphragmatic defect by continuous barbed suture without need to follow the stitches nor to tie knots, (**A**) Taking the first stitch and entering the pre-made loop; (**B**) The first knot took and passed through the pre-made loop; (**C**) Continuous suture without need to follow the stitches; (**D**) Complete closure of the defect with continuous suture without knots.

**Figure 4:**
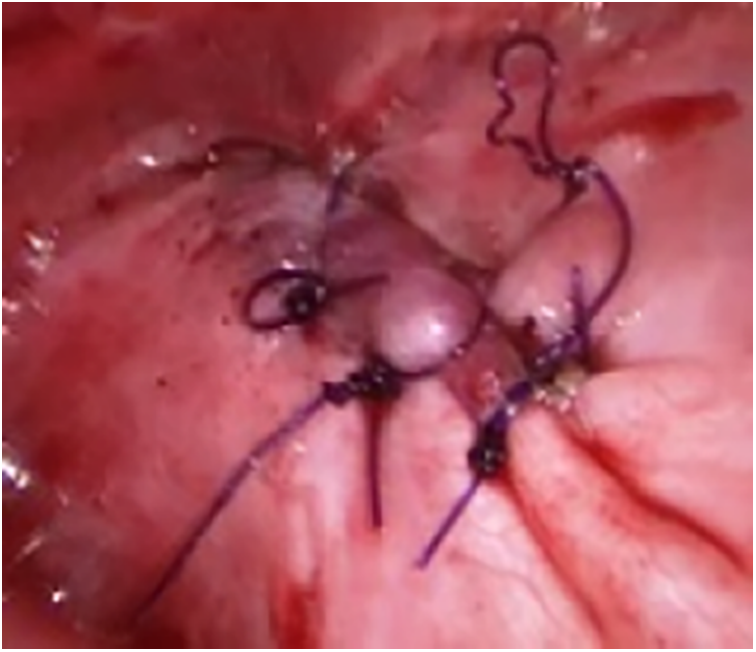
Repair of diaphragmatic repair by interrupted sutures.

The patient was positioned in the lateral decubitus with the head lifted, with a small roll placed under the chest. A 5-mm trocar was inserted in the fourth intercostal space at the tip of the scapula (the patient's upper limb was left free from the body) for a thoracoscope, a second 3 or 5-mm trocar was introduced anteriorly in the fifth or sixth intercostal space below the nipple. A third 3 or 5-mm trocar was introduced posteriorly in the fourth or fifth intercostal space between the optical port and the spine. This changeable position of the working ports helped to meet the ergonomic principle of triangulation. The thoracic cavity was insufflated with CO_2_ at 2–4 mmHg pressure. Following the reduction of the hernia contents into the abdominal cavity, nonabsorbable braided interrupted sutures 2/0 are used to treat the hernia defect with intracorporeal knots or unidirectional nonabsorbable barbed sutures 15 cm in length (V-Loc^TM^ 3.0 and Stratafix^TM^ 2.0 sutures) encompassing two of the diaphragmatic muscle defects (Figures 3, 4).

In case the postero-lateral edge of the diaphragm was deficient, the anterior rim of the diaphragm was sutured directly to the rib by externally tied peri-costal sutures. If a chest tube was needed, it was placed through the posterior trocar port (3).

To facilitate the procedure to deal with the barbed sutures, the anterior working trocar cannula was removed before suturing. The needle holder was passed through the cannula first and continued through the pre-made fixation loop at the end of suture. Then the suture was grasped just close to the needle, and the whole set was passed through the incision made for the anterior trocar (this maneuver was used instead of passing the suture directly through the chest wall as in the non-barbed sutures. It was necessary as the suture loop cannot pass directly through it). After taking the first stitch, we pulled the grasped needle through the fixation loop, which is how the first tie was performed. This suture does not need an assistant to follow during suturing, as it preserves the tension. At the end of suturing, according to the manufacturer's instructions, we took one or two stitches in the opposite direction to suturing and no knots were required.

Intraoperative data were retrieved, including the time needed for reduction, time for excision of the sac, the time required to suture, operation time and size of the defect.

### Postoperative care

Patients started oral feeding as soon as peristalsis was heard. Antibiotics and analgesia were given. In cases with a chest tube, it was removed when a chest x-ray was taken after 24 h and revealed no abnormalities.

Postoperative data were retrieved, including time for full enteral feeding, hospital stay, complications (visceral perforation, wound infection and recurrence), conversion to open and incidence of mortality.

### Statistical analysis

SPSS v26 (IBM Inc., Chicago, IL, USA) was used for statistical analysis. Quantitative variables were presented as mean and standard deviation (SD) and analyzed using the unpaired Student's *t*-test. Qualitative variables were presented as frequency and percentage and analyzed using the Chi-square or Fisher's exact test. A two-tailed *P* value less than or equal to 0.05 was judged statistically significant.

## Results

Preoperative data were insignificantly different in neonatal and delayed presentations between groups B and C (Table 1).

**Table 1:** Preoperative data of the studied groups of neonatal and delayed presentation.

		Neonatal presentation			Delayed presentation		
		Group B (*n* = 43)	Group C (*n* = 55)	*P* value	Group B (*n* = 22)	Group C (*n* = 19)	*P* value
Age		12.7 ± 8.41 (days)	12.96 ± 7.76 (days)	0.871	28.23 ± 17.75 (months)	29.79 ± 15.03 (months)	0.765
Sex	Male	28 (65.12%)	37 (67.27%)	0.823	13 (59.09%)	11 (57.89%)	0.938
	Female	15 (34.88%)	18 (32.73%)		9 (40.91%)	8 (42.11%)	
Weight (kg)		2.91 ± 0.3	3.02 ± 0.31	0.091	13.99 ± 4.16	14.66 ± 2.98	0.565
Side	Right	8 (18.6%)	12 (21.82%)	0.695	3 (13.64%)	2 (10.53%)	0.762
	Left	35 (81.4%)	43 (78.18%)		19 (86.36%)	17 (89.47%)	
Presence of sac		13 (30.23%)	18 (32.73%)	0.792	12 (54.55%)	11 (57.89%)	0.829

Data are presented as mean ± SD or frequency (%).

Contents of hernia were liver in 22 (15.8%) patients. Liver and transverse colon in 5 (3.6%) patients. Omentum, small and large intestine in 33 (23.7%). Stomach, small bowel, large bowel and spleen in 45(32.4%) patients. Omentum and small intestine in 10(7.2%) patients. Stomach, small and large bowel in 6 (4.3%) patients and stomach in 18 (12.9%) patients.

In the neonatal and delayed presentations, the time needed for reduction, time for excision of the sac, and size of defect were insignificantly different between groups B and C.

The mean ± SD of time required to suture was 15.47 ± 3.74 min in group B and 33.93 ± 7.22 min in group C in neonatal presentation and was 13 ± 3.06 min in group B and 28.58 ± 6.75 min in group C in delayed presentation. The mean ± SD of operation time was 94.42 ± 16.59 min in group B and 103.64 ± 24.84 min in group C in neonatal presentation and 83.41 ± 18.09 min in group B and 96.32 ± 20.6 min in group C in delayed presentation. The times required to suture and of operation were significantly shorter in group B than in group C in the neonatal and delayed presentation (*P* value <0.05) (Table 2).

**Table 2:** Intraoperative data of the studied groups of neonatal and delayed presentation.

** **	Neonatal presentation			Delayed presentation		
	Group B (*n* = 43)	Group C (*n* = 55)	*P* value	Group B (*n* = 22)	Group C (*n* = 19)	*P* value
Time needed for reduction (min)	16.84 ± 13.86	18.69 ± 13.83	0.512	14.86 ± 7.49	16.95 ± 6.83	0.360
Time for excision of the sac (min)	6.42 ± 16.73	8.44 ± 20.41	0.601	5.77 ± 5.31	7.05 ± 7.05	0.502
Time required to suture (min)	15.47 ± 3.74	33.93 ± 7.22	<0.001	13 ± 3.06	28.58 ± 6.75	<0.001
Operation time (min)	94.42 ± 16.59	103.64 ± 24.84	0.039	83.41 ± 18.09	96.32 ± 20.6	0.039
Size of the defect (cm^2^)	29.7 ± 16.52	28.11 ± 15.54	0.626	32.68 ± 10.83	36.53 ± 12.34	0.295

Data are presented as mean ± SD.

In the neonatal and delayed presentation, the time for full enteral feeding and hospital stay were insignificantly different between groups B and C (Table 3).

**Table 3:** Postoperative data of the studied groups of neonatal and delayed presentation.

** **	Neonatal presentation			Delayed presentation		
	Group B (*n* = 43)	Group C (*n* = 55)	*P* value	Group B (*n* = 22)	Group C (*n* = 19)	*P* value
Time for full enteral feeding (hours)	31.91 ± 22.31	33.62 ± 23.79	0.717	19.95 ± 5.71	23.42 ± 7.93	0.113
Hospital stay (days)	2.23 ± 0.78	2.11 ± 0.88	0.470	1.82 ± 0.66	2.11 ± 0.81	0.220

Data are presented as mean ± SD.

Complications (visceral perforation, wound infection, and recurrence), conversion to open and mortality were insignificantly different between groups B and C in neonatal CDH. Only wound infection occurs in 3 patients in groups B and C of neonatal CDH. One case exhibited combined visceral perforation and wound infection occurred in group B. One case exhibited combined visceral perforation and recurrence and two cases exhibited combined visceral perforation and wound infection occurred in group C. In the delayed presentation, no patients suffered from major complications in groups B and C (Table 4).

**Table 4:** Complications and mortality of the studied groups of neonatal presentation.

** **		Neonatal presentation		
		Group B (*n* = 43)	Group C (*n* = 55)	*P* value
Complications	Total	7 (16.28%)	5 (9.09%)	0.281
	Visceral perforation	2 (4.65%)	3 (5.45%)	0.858
	Wound infection	3 (6.98%)	3 (5.45%)	0.755
	Recurrence	3 (6.98%)	2 (3.64%)	0.456
Conversion to open		3 (6.98%)	3 (5.45%)	0.755
Mortality		1 (2.33%)	0 (0%)	0.256

Data are presented as frequency (%).

Conversion from a thoracoscopic to open repair occurred in 6 cases (6.1%) owing to bleeding caused by a splenic capsular injury during reduction of contents in a case, splenic capsular injury with injury of the small intestinal mesentery in a case, injury of the mesentery of the small intestine in 2 cases, The fifth case involves intestinal injury and perforation caused by instrument manipulation. In the 6th case; a 1-day-old infant had a large defect with nearly all abdominal contents in the chest, making it difficult to reduce them and complete the technique. Laparotomy was performed through a left subcostal incision, after reducing the contents in the abdomen, primary repair of the defect was performed with a tube drain in the left upper quadrant. The course of recovery was unremarkable 42.57 + 13.04.

Regular follow-up of the studied groups was 2.5 ± 1.08 year. Follow-up of neonatal participants showed that recurrence occurred in 5 of 92 cases in which thoracoscopic repair was completed (5.4%). The recurrence occurred 3 months later in 2 cases, one after 4 months, and 2 cases discovered after 1 year. Plain x-rays of the chest and abdomen, as well as contrast gastrointestinal radiography, were used to make their diagnosis. Parents refused thoracoscopic repair, so a left subcostal incision was made for an open approach. During laparotomy, partial dehiscence of the repair was discovered and primarily repaired by releasing the posterior leaflet in the two cases discovered after one year without tension. Early cases of recurrence were due to repair under tension and needed patch repair as the release was insufficient.

Mortality occurred only in a neonate aged 1-day postoperative due to disseminated intravascular coagulation (DIC).

## Discussion

MIS is safe and beneficial for newborns and pediatric patients (13). Suturing tissues is one of advanced MIS's most challenging and time-consuming jobs, despite attempts to promote techniques and training (14). Thoracoscopic repair may improve clinical outcomes among pediatrics with CDH. Nevertheless, the restricted field and significant diaphragmatic defects in neonates are barriers (15).

Intracorporeal knot tying is effective and involves no specialized instruments, but even so, it needs a high level of surgical talent and lengthens the duration of the operation. Considering that neonates with CDH are particularly susceptible to adverse perioperative incidents, the capacity to conserve time during the operation is crucial. In contrast, extracorporeal knot tying is widespread and can be performed with various tools, such as the TI-KNOT system (LSI Systems). However, these special instruments may lack availability or inaccessibility in some regions with scarce resources (15).

Extracorporeal knot tying with a pusher in the repair of CDH in pediatrics is time-consuming as the needle cannot be introduced or extracted through the small sized cannulas 3 or 5 mm, so with each stitch, you had to pull out the cannula from the chest wall and insert the needle through the chest opening and then insert the cannula again, the same during retrieval of the needle after taking the suture, and to be repeated as much as stitches will be taken. Even if the needle is inserted through the chest wall, after taking the stitch, the needle needs to be retrieved from chest opening and to pull such a long two ends of the thread (compared to very small chest space in neonates) through the chest opening of the trocar site. This will lead to enlarging the opening more than the cannula and more air leakage (even if the work was without a trocar, as some do, and the instrument inserted in the chest without trocars), prolonging the operative time. Besides, the extracorporeal tie is weaker than the intracorporeal one (16).

The unidirectional barbed sutures are a new suture designed to minimize operative times by eliminating the requirement for knots during MIS suturing (9). Barbed sutures have been extensively utilized in MIS on adults and were effective, safe, and time-saving (17). Using unidirectional barbed sutures for repairing diaphragmatic hernias has emerged based on studies demonstrating their effectiveness in children undergoing laparoscopic gastrointestinal reconstruction (12).

Comparing the mean tensile strengths of five types of sutures (barbed and non-barbed), demonstrated that strength varied between these categories. Regarding stiffness and elongation, barbed sutures differed significantly from the other non-barbed sutures, as determined by pairwise comparisons. The strength of the barbed sutures was intermediate between that of 2.0 and 3.0 non-barbed sutures and did not differ significantly from 3.0 non-barbed sutures. Elongation of barbed sutures was nearest to that of 3.0 non-barbed sutures; barbed suture stiffness was significantly greater than any other suture type. The intra-category variation in strength and stiffness was significantly greater for barbed sutures than for any other type of suture (18).

The breaking strength of barbed 2.0 polypropylene sutures appears to be intermediate between that of 2.0 and 3.0 non-barbed sutures and is closer to that of 3.0 non-barbed sutures. In addition, barbed sutures seem significantly stiffer than non-barbed sutures (18).

The bidirectional tensile pull strength of the barbed suture is identical to that of a non-barbed suture of the same material but one size smaller. In straight tensile testing, a 2-0 barbed suture was as powerful as a 3-0 non-barbed suture of a similar substance (18). The suture was constructed to fix itself to each 1 mm of tissue and can be positioned using typical intracorporeal procedures (19).

This article is about using barbed sutures in the thoracoscopic repair of CDH in pediatrics. Several modified techniques have been advocated to facilitate the minimal access procedure. Here, we aimed to simplify the thoracoscopic repair of CDH by applying readily available barbed knot-tying sutures. We used V-Loc^TM^ 3/0 from Covidien and Stratafix^TM^2/0 sutures From Johnson & Johnson, each 15 cm in length (according to availability); according to the manufacturers, 2/0 Stratafix^TM^ is equal to 3/0 V-Loc^TM^. We used the 15 cm length as it is the least available length that can be easier to be handled in the small thoracic cavity, as other available lengths, 30 and 45 cm, are very long to be handled in our situation.

Using the V-LOC^TM^, the unique notion of a flowing suture line to repair a hernia defect is a logical and practical procedure that is straightforward to perform. The objective was to shorten operation time and simplify the technique, decreasing the dangers of prolonged anaesthesia and CO_2_ exposure. The unidirectional barbed sutures are easily managed, are effortless to use, and result in drastically reduced operative times than conventional interrupted intracorporeal knots in hernia defect repair surgery (20).

Utilization of barbed sutures considerably decreased the time of suturing required to correct uterine wall abnormalities and the total operational time compared with traditional sutures in adults (21). Additionally, barbed suture considerably aids laparoscopic myomectomy by decreasing overall operative/suturing time, as concluded in the meta-analysis by Gardella et al. (9).

Utilization of the unidirectional knotless barbed suture in pediatric surgery was assessed by Lukish et al. (22). They highlighted that V-LOC barbed suture is a new, safe, and time-saving alternative for pediatric MIS. Two (18.18%) infants with Bochdalek CDH suffered a recurrence between the ages of 4 and 6 months and needed redo surgery. The remaining nine children had no more difficulties or recurrences and no fatalities.

Our results are supported by another report by Lukish et al. (12). They reported in their pilot study that V-LOC^TM^ (a novel continuous stitch laparoscopic fundoplication) resulted in a thirty percent reduction in operative time than standard laparoscopic Nissen fundoplication (79.1 ± 24.2 min vs. 113.8 ± 25.9 min respectively, *P* < 0.05). Although the follow-up was short, the V-LOC continuous stitch laparoscopic fundoplication is a safe and successful method that can potentially shorten the operation time in children with gastroesophageal reflux syndrome. This approach may be useful in additional minimally invasive pediatric surgical applications.

In pediatrics, the shorter the operation time, the lower risk for complications. As numerous studies have hypothesized and observed relationships between longer anaesthetic time and problems such as postoperative nausea and vomiting (23), surgical site infection (24), hypothermia (25), cardiopulmonary problems, and mortality (26).

Our results indicated that operative time in delayed group is shorter than neonatal group and this can be explained due to relatively larger cavity in the delayed group which allow more space for handling instruments and suture.

Our retrospective analysis approach is a limitation of this study. Therefore, further prospective randomized clinical trials with longer follow-ups are required to validate our findings.

## Conclusions

Both unidirectional barbed sutures and intracorporeal knots were safe and effective, however, unidirectional barbed sutures are innovative and timesaving for CDH thoracoscopic repair in early and late presentations.

## Data Availability

The raw data supporting the conclusions of this article will be made available by the authors, without undue reservation.
